# Association of appendicular skeletal muscle mass with carotid intima-media thickness according to body mass index in Korean adults

**DOI:** 10.4178/epih.e2018049

**Published:** 2018-10-07

**Authors:** Ji Eun Heo, Hyeon Chang Kim, Jee-Seon Shim, Bo Mi Song, Hye Yoon Bae, Ho Jae Lee, Il Suh

**Affiliations:** 1 Department of Public Health, Yonsei University Graduate School, Seoul, Korea; 2 Cardiovascular and Metabolic Disease Etiology Research Center, Yonsei University College of Medicine, Seoul, Korea; 3 Department of Preventive Medicine, Yonsei University College of Medicine, Seoul, Korea

**Keywords:** Sarcopenia, Muscles, Skeletal, Body mass index, Body composition, Atherosclerosis

## Abstract

**OBJECTIVES:**

The combined effects of obesity and appendicular skeletal muscle (ASM) on atherosclerosis, especially in middleaged populations, remain poorly understood. This cross-sectional study investigated the effects of ASM on carotid intima-media thickness (IMT) according to body mass index (BMI) in middle-aged Korean adults.

**METHODS:**

Herein, 595 men and 1,274 women aged 30-64 years completed questionnaires and underwent health examinations as part of the Cardiovascular and Metabolic Disease Etiology Research Center cohort. ASM was measured via bioelectrical impedance analysis and adjusted for weight (ASM/Wt). IMT was assessed using B-mode ultrasonography; highest quartile of IMT was defined as gender-specific top quartile of the IMT values. Higher BMIs was defined as a BMI over 25.0 kg/m^2^ .

**RESULTS:**

Compared to the highest ASM/Wt quartile, the lowest ASM/Wt quartile was significantly associated with highest quartile of IMT in men with lower BMIs (adjusted odds ratio [aOR], 2.78; 95% confidence interval [CI], 1.09 to 7.13), but not in those with higher BMIs (aOR, 0.59; 95% CI, 0.24 to 1.91). In women, there was no significant association of low skeletal muscle mass with highest quartile of IMT, regardless of BMI.

**CONCLUSIONS:**

Low appendicular skeletal muscle mass is associated with carotid arterial wall thickening in men with lower BMIs, but not in men with higher BMIs. Our findings suggest that the risk of atherosclerosis may be low in middle-aged Korean men with appropriate body weight and skeletal muscle mass maintenance.

## INTRODUCTION

Although obesity is a major modifiable risk factor for cardiovascular disease (CVD), the mechanisms linking obesity and atherosclerosis are not fully understood. Sarcopenia, an age-related decline in skeletal muscle mass, leads to metabolic and vascular abnormalities [1,2]. Previous epidemiologic studies of the combined effects of low muscle mass and obesity on cardiometabolic disorders have yielded inconsistent results [3-8]. Moreover, most of these studies screened for the presence of a synergistic relationship between muscle mass and body mass index (BMI) in older adults. Little information is available regarding the independent relationship between skeletal muscle mass and atherosclerosis in the middle-aged population. Furthermore, few studies have investigated whether associations between skeletal muscle mass and arterial wall thickness may vary according to obesity categories. The purpose of this study was to evaluate the association of skeletal muscle mass with 2 markers of atherosclerosis—a higher carotid intima-media thickness (IMT) and carotid artery plaque—in a middle-aged Korean population stratified by BMI categories.

## MATERIALS AND METHODS

### Study population

The present study used data from the Cardiovascular and Metabolic Disease Etiology Research Center cohort study, which was designed to recruit members of the general population residing in 4 districts (Seoul, Goyang, Gimpo, and Incheon) in South Korea (hereafter Korea). The eligibility criteria for inclusion were being aged 30 to 64 years, having lived for more than 8 months of the year at the current residence with no plans to move over the next 2 years, and having the ability to provide verbal or written consent to participate in the study. Participants were excluded if they had been diagnosed with cancer within the last 2 years or were currently being treated for cancer; had a history of myocardial infarction, stroke, or heart failure; were currently involved in pharmaceutical trials; or were currently pregnant or reported the possibility of being pregnant on the day of registration. The sampling and measurement procedures have been described in detail previously [9]. This study initially enrolled 2,465 participants aged 30-64 years between 2013 and 2015. All participants completed health questionnaires and examinations according to a predefined protocol. People aged <40 years (n=551), those with missing key variables (n=14), and those with a BMI under 18.5 kg/m^2^ (n=31) were excluded from the current analyses. Finally, a cross-sectional analysis of the remaining 595 men and 1,274 women was conducted. All participants provided written informed consent, and the study protocol was approved by the institutional review board hospital at Yonsei University College of Medicine (4-2013-0661).

### Questionnaire data

All participants were individually interviewed using standardized questionnaires to obtain information regarding demographics, medication use, and lifestyle behaviors. Trained interviewers conducted face-to-face interviews and questionnaire surveys according to the protocol. Three groups were stratified by smoking status: current smokers, former smokers, and non-smokers. Similarly, participants were classified as current regular alcohol drinkers, former drinkers, or non-drinkers. Physical activity was assessed using the International Physical Activity Questionnaire-Short Form. Regular exercise was defined as moderate- to high-intensity physical activity performed at least 3 times per week.

### Physical examination

Standing height was measured to the nearest 0.1 cm using a stadiometer (DS-102, Dong Sahn Jenix, Seoul, Korea), and body weight was measured to the nearest 0.1 kg on a digital scale (DB-150, CAS, Seongnam, Korea) according to the predetermined protocol. BMI was calculated as the body weight divided by the standing height squared (kg/m^2^). Waist circumference was assessed to the nearest 0.1 cm at the midpoint between the lower border of the rib cage and the iliac crest using an ergonomic circumference-measuring tape (Seca 201, Seca, Hamburg, Germany). Participants rested for ≥5 minutes before blood pressure measurements were made, and systolic and diastolic blood pressures were measured 3 times at 2-minute intervals. The average of last 2 measurements was used in the analysis.

### Laboratory assays

Blood samples were collected from the antecubital vein after the patients had fasted for at least 8 hours. Total cholesterol (TC), high-density lipoprotein cholesterol (HDLC), low-density lipoprotein cholesterol, and triglyceride levels were measured via enzymatic methods (Advia 1800 autoanalyzer, Siemens Medical Solutions, Deerfield, IL, USA). The TC to HDLC ratio (TC/HDLC) was calculated to evaluate dyslipidemia. Fasting blood glucose concentrations were measured using a colorimetric method (Advia 1800 autoanalyzer, Siemens Medical Solutions), and hemoglobin A1c (HbA1c) concentrations were assessed via high-performance liquid chromatography (Variant II Turbo, Bio-Rad, Berkeley, CA, USA) according to the National Glycohemoglobin Standardization Program guidelines. C-reactive protein concentrations were determined using a turbidimetric immunoassay (Advia 1800 autoanalyzer, Siemens Medical Solutions).

### Measurement of skeletal muscle mass

Appendicular skeletal muscle mass (ASM) was measured via bioelectrical impedance analysis (BIA) using Inbody370 (Biospace, Seoul, Korea) according to the instructions provided by the manufacturer. ASM was determined as the sum of the lean muscle masses of both arms and both legs. As the absolute amount of skeletal muscle is known to correlate strongly with body size, ASM is commonly adjusted using indicators of body size such as the height squared, weight, or BMI. In this study, we used weight-adjusted ASM (ASM/Wt) because previous studies reported that this parameter correlated better with cardiometabolic risk factors than did height squared- or BMI-adjusted ASM [10,11]. Participants were divided into 4 groups based on gender-specific ASM/Wt quartiles: <30.85, 30.85-32.36, 32.37-33.87, and ≥33.88% for men and <25.84, 25.84-27.31, 27.32-28.90, and ≥28.91% for women.

### Measurement of arterial wall thickness

The bilateral common carotid arteries were assessed via B-mode ultrasonography with an 8-MHz linear probe (Accuvix XG, Samsung Medison, Seoul, Korea) according to a predetermined protocol. Participants were placed in the supine position with the head turned at a 30° angle contralateral to the scanning side. Subsequently, the IMT was measured bilaterally in the 1-cm segment proximal to the carotid bulb dilatation and calculated as the mean value of the right and left common carotid arteries from computer-based measurement points in the region. The highest quartile of IMT was defined as the gender-specific top quartile of the mean IMT value (≥0.755 mm for men and ≥0.724 mm for women). Carotid artery plaque was defined as a focal thickening exceeding 1.0 mm or ≥50% thicker than that of the surrounding vessel wall.

### Statistical analysis

All analyses were performed separately for men and women because both ASM/Wt and IMT differed significantly by gender (Supplementary Material 1). We evaluated differences in demographic characteristics among the 4 groups based on the ASM/Wt quartiles and used 1-way analysis of variance for normally distributed variables, the Kruskal-Wallis test for skewed variables, and the chi-square test for categorical variables. The p for trend was calculated using a contrast to test for linear trends in continuous variables and the Cochran-Armitage test for categorical variables. The association between ASM/Wt and the highest quartile of IMT was assessed in people with lower (<25.0 kg/m^2^) and higher BMIs (≥25.0 kg/m^2^) because there was a significant interaction between ASM/Wt quartiles and BMI categories in the presence of the highest quartile of IMT (Supplementary Material 2). Multivariable logistic regression analyses were used to assess the independent effect of ASM/Wt (using gender-specific quartiles) on the likelihood of being in highest quartile of IMT according to BMI categories in both age-adjusted and fully adjusted models (age, menopause, systolic blood pressure, HbA1c, TC/HDLC, C-reactive protein, smoking, drinking, and regular exercise). ASM/Wt was also analyzed as a continuous variable in association with the highest quartile of IMT. Additionally, the same multivariable logistic regression analyses were conducted to determine the association between ASM/Wt (using gender-specific quartiles and continuous variables) and carotid artery plaque according to BMI categories. Lastly, we estimated the odds ratio (OR) and 95% confidence interval (CI) for being in the highest quartile of IMT according to the 8 combined categories of ASM/Wt (quartiles) and BMI (<25.0 or ≥25.0 kg/m^2^) after fully adjusting for confounders. The reference group comprised participants with lower BMIs in the highest ASM/Wt quartile. All statistical tests were performed using SAS version 9.4 (SAS Institute Inc., Cary, NC, USA), and statistical significance was defined as a 2-sided p-value of <0.05.

## RESULTS

Table 1 presents the characteristics of the men and women study participants according to ASM/Wt quartiles. Both men and women in the lower ASM/Wt quartiles tended to have a higher BMI, waist circumference, blood pressure, lipid levels, glucose level, IMT, frequencies of the highest quartile of IMT, and carotid artery plaque. However, higher ASM/Wt quartiles were not significantly associated with smoking, drinking status, or sleep duration in either gender.

Table 2 presents the results of multivariable logistic regression analyses stratified by BMI categories. In the age-adjusted logistic regression model, the risk of being in the highest quartile of IMT among men with lower BMIs was significantly higher in the lowest ASM/Wt quartile (OR, 3.21; 95% CI, 1.32 to 7.82) relative to the highest ASM/Wt quartile. After adjusting for age, systolic blood pressure, HbA1c, TC/HDLC, C-reactive protein, smoking, drinking, and regular exercise, this association was weakened but remained statistically significant (OR, 2.78; 95% CI, 1.09 to 7.13). When continuous variables were examined in the age-adjusted model, each 10% decrease in ASM/Wt (OR, 4.32; 95% CI, 1.12 to 16.71) was inversely associated with being in the highest quartile of IMT in men with lower BMIs. After adjusting for additional CVD risk factors, the association became non-significant. Among men with higher BMIs, the risk of being in the highest quartile of IMT was lower in other ASM/Wt quartiles than in the highest ASM/Wt quartile, although this association was not significant. Among women, there was no significant association between ASM/Wt and being in the highest quartile of IMT, regardless of BMI category.

The results for using carotid artery plaque as an outcome variable were similar to those of using the highest quartile of IMT. After adjusting for CVD risk factors, the risk of carotid artery plaque among men with lower BMIs was significantly higher in the lowest ASM/Wt quartile (OR, 2.48; 95% CI, 1.04 to 5.90) than in the highest ASM/Wt quartile. Among men with higher BMIs, the risk of carotid artery plaque was lower in other ASM/Wt quartiles than in the highest ASM/Wt quartile, although this association was not significant. Among women, there was no significant association between ASM/Wt and carotid artery plaque, regardless of BMI category (Table 3).

**Figure 1.**
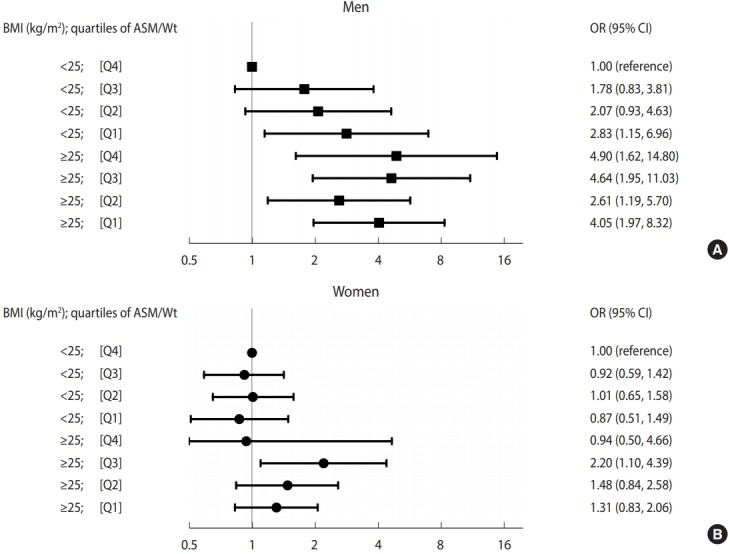
ORs for higher quartile of IMT according to the eight combined categories of ASM/Wt and BMI by gender (A: men, B: women). ORs (95% CIs) adjusted for age, menopause, systolic blood pressure, hemoglobin A1c, total/high-density lipoprotein cholesterol ratio, smoking, drinking, and regular exercise are shown. The reference group comprised individuals with a BMI of <25 kg/m^2^ and an ASM/Wt in the highest quartile. BMI, body mass index; ASM/Wt, appendicular skeletal muscle mass/weight; OR, odds ratio; CI, confidence interval; IMT, intima-media thickness.

We estimated the OR for being in the highest quartile of IMT according to the 8 combined categories of ASM/Wt and BMI (Figure 1). The reference group consisted of those with lower BMIs in the highest ASM/Wt quartile. Men with lower BMIs in the lowest ASM/Wt quartile had a 2.83-fold higher risk of being in the highest quartile of IMT (95% CI, 1.15 to 6.96) than the reference group. Men with higher BMIs had significantly higher ORs for being in the highest quartile of IMT than the reference group, regardless of the ASM/Wt quartile (quartile [Q] 1: 4.05, Q2: 2.61, Q3: 4.64, Q4: 4.90). No association between ASM/Wt and the highest quartile of IMT was observed among women with lower BMIs. However, women with higher BMIs in the third ASM/Wt quartile had a 2.20-fold higher risk of being in the highest quartile of IMT (95% CI, 1.10 to 4.39) than the reference group.

## DISCUSSION

The current study found that low skeletal muscle mass was independently associated with the highest quartile of IMT and carotid artery plaque in a cohort of middle-aged Korean men with lower BMIs, but not in those with higher BMIs. In women, there was no significant association of low skeletal muscle mass with the highest quartile of IMT or carotid artery plaque, regardless of BMI.

Several studies reporting inverse associations between the skeletal muscle mass and subclinical atherosclerosis have presumed that these phenomena may share a similar pathway and thus facilitate mutual abnormalities [12-15]. Moreover, several studies have examined the differential effects of various skeletal muscle mass and BMI categories on the risks of CVD or metabolic disorders [3-7], as skeletal muscle mass and obesity are not independent factors. One study reported that individuals with lower skeletal muscle mass and a lower BMI were at high risk [6], whereas others reported that those with lower skeletal muscle mass and a higher BMI were at high risk [3,4]. Still other studies found that individuals with lower skeletal muscle mass were at high risk for CVD or hypertension, regardless of BMI [5,7]. In contrast, some studies did not find any independent associations between skeletal muscle mass and subclinical atherosclerosis [16,17].

Although the mechanism underlying the potential association between low skeletal muscle mass and arterial wall thickening is unclear, both a decrease in skeletal muscle mass and an increase in arterial wall thickness may be age-related. Most previous studies about muscle mass, atherosclerosis, and CVD have mainly evaluated elderly populations [3,4,7,13,18,19]. Only a few studies have suggested that the inverse relationship between skeletal muscle mass and CVD risk was significant not only for elderly individuals, but also for middle-aged individuals [5,20]. In the current study, we also found an independent association between low skeletal muscle mass and arterial wall thickening in middle-aged non-obese men. To investigate whether there were different associations according to young and old age, we performed an additional analysis stratified by age subgroups (age ≤55 vs. >55 years). However, we were not able to examine the direct effect of age on the association between low skeletal muscle mass and arterial wall thickening (data not shown).

In this study, according to the stratified analysis (Table 2), the inverse association of skeletal muscle mass with arterial wall thickness was significant only in men with lower BMIs, but not in those with higher BMIs. In the combined analysis (Figure 1), we used a single reference group that consisted of non-obese men with the highest quartile of skeletal muscle mass. Non-obese men had a gradually higher risk of arterial wall thickening as skeletal muscle mass decreased compared with the reference group. However, obese men had a significantly higher risk of arterial wall thickening, regardless of skeletal muscle mass, compared with the reference group. Currently, the mechanisms underlying the differential relationship of skeletal muscle mass and arterial wall thickness according to BMI status remain unclear, although the presence of inflammatory markers might partially explain the observed outcomes. Previous animal and human studies have suggested associations of low-grade inflammation with low skeletal muscle mass [21], obesity [22], and atherosclerosis [23], although these relationships are not fully understood. In the current study, obese men regardless of skeletal muscle mass and non-obese men with low skeletal muscle mass had low-grade inflammation (data not shown), which may have affected arterial wall thickening. Previous studies have suggested that physical activity [24] and insulin resistance [18] might also exhibit differential relationships with skeletal muscle mass decline and arterial wall thickening according to obesity. To determine whether insulin resistance played a role in the association between low skeletal muscle mass and arterial wall thickening, we additionally adjusted for the insulin resistance index (data not shown); this caused the effect of low skeletal muscle mass to diminished to borderline in men with lower BMIs, suggesting that insulin resistance may partially but not fully explain the association between low appendicular skeletal muscle mass and arterial wall thickening.

As we mentioned, the significant relationship between low skeletal muscle mass and the highest quartile of IMT was not observed in women. Although the mechanism is unclear, there are some possible explanations. First, biological differences between genders might modify the effect of skeletal muscle mass on arterial wall stiffness or thickness. Age-related declines in skeletal muscle mass and muscle strength affect both genders, but are more prominent in men than in women [25]. Second, gender differences can also be attributed to gender-specific effects of sex hormones. In men, changes in skeletal muscle mass are controlled by testosterone levels, whereas women have much lower absolute degrees of decline in testosterone relative to men and therefore do not experience the effects of testosterone on skeletal muscle mass. Female sex hormones, especially estrogen, provide protective effects on cardiovascular function by directly modulating the renin-angiotensin-aldosterone system [26,27]. Lastly, different lifestyle factors between genders, such as smoking status and alcohol intake, could affect the relationship between skeletal muscle mass and arterial wall thickness. In this aspect, women would likely be less affected by skeletal muscle mass declines than men. This finding is consistent with those of a few previous reports [12,15].

The current study also had some limitations of note. First, this study was cross-sectional in nature; therefore, the causal association between low muscle mass and arterial wall thickening is uncertain. Second, our estimation of skeletal muscle mass did not involve highly accurate modalities such as dual-energy X-ray absorptiometry (DEXA), computed tomography, or magnetic resonance imaging. However, BIA is a non-invasive method for assessing skeletal muscle mass that is useful in large population-based studies. Previous studies have reported a good linear correlation of the BIA and DEXA methods for estimating skeletal muscle mass in healthy populations, regardless of gender, age, and race [28-30]. One such study of healthy men and women reported strong correlations of body composition parameters (correlation coefficients, 0.82 to 0.95) measured using BIA and DEXA [29]. However, a weakness of the BIA method is the potential effect of the participant’s hydration status. Accordingly, we asked our participants to fast for at least 8 hours to reduce the possibility of measurement errors. Finally, BMI has limitations as an index of obesity, since it reflects not only fat mass, but also muscle mass.

In conclusion, we observed associations of low appendicular skeletal muscle mass with high IMT and carotid artery plaque in men with lower BMIs, but not in those with higher BMIs, even after adjusting for traditional cardiovascular risk factors. In women, there was no significant association of low appendicular skeletal muscle mass with high IMT and carotid artery plaque, regardless of BMI. Our findings suggest that the risk of atherosclerosis may be low in middle-aged Korean men with an appropriate body weight and skeletal muscle mass maintenance. Further prospective studies are needed to confirm that increasing skeletal muscle mass could decrease the risk of atherosclerosis in middle-aged Korean men with an appropriate body weight.

## Figures and Tables

**Table 1.** Characteristics of gender stratified by ASM/Wt quartiles

Variables	Quartiles of ASM/Wt											
	Men (n=595)				p for difference^1^	p for trend^2^	Women (n=1,274)				p for difference^1^	p for trend^2^
	Q1 (<30.85)	Q2 (30.85–32.36)	Q3 (32.37–33.87)	Q4 (≥33.88)			Q1 (<25.84)	Q2 (25.84–27.31)	Q3 (27.32–28.90)	Q4 (≥28.91)		
Age (yr)	55.0±6.6	55.0±6.3	54.4±6.6	53.9±6.8	0.38	0.10	55.8±5.4	55.0±5.9	53.9±6.1	52.2±6.1	<0.001	<0.001
Height (cm)	167.2±5.7	169.0±5.4	170.4±5.4	171.2±6.0	<0.001	<0.001	154.1±4.6	156.4±4.8	158.0±4.5	159.5±5.0	<0.001	<0.001
Weight (kg)	75.3±10.9	71.8±8.4	69.6±7.8	66.3±8.4	<0.001	<0.001	62.4±8.6	58.7±7.0	57.3±6.3	54.8±5.6	<0.001	<0.001
BMI (kg/m^2^)	26.8±2.6	25.1±2.1	23.9±1.8	22.5±2.0	<0.001	<0.001	26.2±2.9	24.0±2.2	22.9±1.9	21.5±1.7	<0.001	<0.001
Waist circumference (cm)	92.1±7.3	87.0±5.9	84.4±5.5	80.4±6.1	<0.001	<0.001	84.4±8.0	79.3±6.6	77.0±6.0	73.1±5.5	<0.001	<0.001
ASM (kg)	22.1±3.0	22.7±2.6	23.1±2.6	23.7±3.4	<0.001	<0.001	15.3±2.0	15.6±1.9	16.1±1.8	16.8±2.2	<0.001	<0.001
Systolic BP (mmHg)	128.7±14.8	125.0±14.9	124.9±13.1	120.9±13.5	<0.001	<0.001	119.5±13.1	117.8±13.8	114.6±13.6	111.1±14.3	<0.001	<0.001
Diastolic BP (mmHg)	82.5±10.1	79.8±9.5	80.7±9.4	78.1±9.9	0.001	<0.001	75.3±8.5	74.2±8.8	73.0±8.8	71.2±8.7	<0.001	<0.001
TC (mg/dL)	200.6±35.4	196.2±33.6	194.2±37.3	191.0±31.0	0.11	0.01	205.5±35.9	201.3±35.0	205.1±35.3	195.9±31.9	0.001	0.002
HDLC (mg/dL)	48.6±10.3	48.8±11.3	52.0±13.6	56.5±14.2	0.001	<0.001	56.8±13.3	58.3±13.7	60.3±13.7	63.8±14.9	<0.001	<0.001
LDLC (mg/dL)	127.3±32.1	120.1±31.5	121.0±33.3	111.8±27.4	0.005	<0.001	124.8±31.7	121.4±31.7	123.2±29.9	113.0±28.0	<0.001	<0.001
TG (mg/dL)	150 [107-204]	135 [96-204]	124 [93-162]	105 [73-136]	<0.001	<0.001	116 [91-160]	108 [82-141]	100 [75-138]	86 [66-117]	<0.001	<0.001
TC/HDLC	4.3±0.9	4.2±1.0	3.9±1.0	3.6±1.0	<0.001	<0.001	3.8±0.9	3.6±0.8	3.5±0.8	3.2±0.8	<0.001	<0.001
Fasting glucose (mg/dL)	97 [87-106]	90 [84-97]	90 [85-98]	92 [85-100]	<0.001	<0.001	90 [84-98]	88 [83-94]	87 [82-93]	86 [81-91]	<0.001	<0.001
Hemoglobin A1c (%)	6.0±1.1	5.7±0.8	5.6±0.5	5.7±0.6	0.001	<0.001	5.9±0.8	5.8±0.6	5.7±0.6	5.6±0.6	<0.001	<0.001
C-reactive protein (mg/L)	1.1 [0.6-1.7]	0.7 [0.4-1.2]	0.6 [0.3-1.1]	0.5 [0.3-1.0]	0.28	0.20	0.9 [0.5-2.0]	0.6 [0.4-1.2]	0.5 [0.3-0.9]	0.4 [0.3-0.8]	0.004	<0.001
IMT (mm)	0.711±0.115	0.681±0.099	0.672±0.110	0.662±0.097	0.001	<0.001	0.678±0.095	0.669±0.105	0.657±0.098	0.632±0.100	<0.001	<0.001
Highest quartile of IMT	54 (36.5)	36 (24.2)	37 (24.8)	23 (15.4)	<0.001	<0.001	96 (30.2)	90 (28.2)	77 (24.1)	58 (18.2)	0.003	<0.001
Carotid plaque	44 (29.5)	36 (24.2)	30 (20.1)	28 (18.9)	0.12	0.02	45 (14.2)	40 (12.6)	29 (9.1)	23 (7.2)	0.019	<0.001
Antihypertensive medication	47 (31.8)	39 (26.2)	28 (18.8)	20 (13.4)	<0.001	<0.001	73 (23.0)	66 (20.7)	39 (12.2)	29 (9.1)	<0.001	<0.001
Antidiabetic medication	17 (11.5)	11 (7.4)	8 (5.4)	9 (6.0)	0.22	0.06	23 (7.2)	10 (3.1)	9 (2.8)	8 (2.5)	0.006	0.003
Lipid-lowering medication	22 (14.9)	15 (10.1)	15 (10.1)	9 (6.0)	0.11	0.02	57 (17.9)	66 (20.7)	38 (12.0)	22 (6.9)	<0.001	<0.001
Smoking status												
Non-smoker	35 (23.7)	29 (19.5)	41 (27.5)	32 (21.5)	0.34	0.10	302 (95.0)	304 (95.3)	304 (95.3)	297 (93.4)	0.28	0.02
Former smoker	76 (51.3)	77 (51.7)	65 (43.6)	66 (44.3)			13 (4.1)	8 (2.5)	8 (2.5)	9 (2.8)		
Current smoker	37 (25.0)	43 (28.8)	43 (28.9)	51 (34.2)			3 (0.9)	7 (2.2)	7 (2.2)	12 (3.8)		
Drinking status												
Non-drinker	18 (12.2)	17 (11.4)	19 (12.8)	13 (8.7)	0.55	0.72	133 (41.9)	122 (38.2)	134 (42.0)	116 (36.5)	0.76	0.30
Former drinker	7 (4.7)	13 (8.7)	14 (9.4)	9 (6.1)			10 (3.1)	12 (3.8)	9 (2.8)	10 (3.1)		
Current regular drinker	123 (83.1)	119 (79.9)	116 (77.8)	127 (85.2)			175 (55.0)	185 (58.0)	176 (55.2)	192 (60.4)		
Regular exercise												
Yes	85 (57.4)	105 (70.5)	90 (60.4)	106 (71.1)	0.02	0.09	186 (58.5)	198 (62.1)	202 (63.3)	208 (65.4)	0.32	0.07
No	63(42.6)	44(29.5)	59(39.4)	43(28.9)			132 (41.5)	121 (37.9)	117 (36.7)	110 (34.6)		
Sleep duration (hr/d)	7.1±1.3	6.9±1.3	7.1±1.1	6.8±1.1	0.09	0.11	6.8±1.3	6.9±1.1	6.8±1.2	6.7±1.2	0.40	0.46
Menopause							264 (83.0)	247 (77.4)	227 (71.2)	187 (58.8)	<0.001	<0.001

Values are presented as mean±standard deviation, median [interquartile range], or number (%). ASM/Wt, appendicular skeletal muscle mass/weight; BMI, body mass index; BP, blood pressure; TC, total cholesterol; HDLC, high-density lipoprotein cholesterol; LDLC, low-density lipoprotein cholesterol; TG, triglycerides; IMT, intima-media thickness.

1 Using 1-way analysis of variance, the Kruskal–Wallis test, or the chi-square test.

2 From a general linear model using contrast coefficients or the Cochran-Armitage trend test.

**Table 2.** Association between ASM/Wt and the highest quartile of IMT according to BMI categories

	No. of people	People with the highest quartile of IMT	Age-adjusted	Fully adjusted^1^
Men (n=595)				
BMI <25 kg/m^2^				
ASM/Wt (quartiles)				
Q4	126	15 (11.9)	1.00 (reference)	1.00 (reference)
Q3	102	20 (19.6)	1.78 (0.84, 3.79)	1.59 (0.72, 3.50)
Q2	75	17 (22.7)	2.04 (0.92, 4.52)	1.81 (0.78, 4.17)
Q1	36	14 (38.9)	3.21 (1.32, 7.82)	2.78 (1.09, 7.13)
ASM/Wt (continuous)				
Per 10% decrease	339	66 (19.5)	4.32 (1.12, 16.71)	3.26 (0.78, 13.68)
BMI ≥25 kg/m^2^				
ASM/Wt (quartiles)				
Q4	23	8 (34.8)	1.00 (reference)	1.00 (reference)
Q3	47	17 (36.2)	0.99 (0.35, 2.76)	0.91 (0.25, 2.96)
Q2	74	19 (25.7)	0.56 (0.19, 1.65)	0.42 (0.14, 1.34)
Q1	112	40 (35.7)	0.99 (0.35, 2.76)	0.59 (0.24, 1.91)
ASM/Wt (continuous)				
Per 10% decrease	256	84 (32.8)	1.27 (0.48, 3.35)	0.82 (0.30, 2.22)
Women (n=1,274)				
BMI <25 kg/m^2^				
ASM/Wt (quartiles)				
Q4	307	56 (18.3)	1.00 (reference)	1.00 (reference)
Q3	271	58 (21.4)	1.01 (0.66, 1.55)	0.90 (0.57, 1.42)
Q2	225	56 (24.9)	1.10 (0.71, 1.70)	0.96 (0.62, 1.52)
Q1	122	30 (24.6)	0.95 (0.56, 1.61)	0.85 (0.49, 1.47)
ASM/Wt (continuous)				
Per 10% decrease	925	200 (21.6)	1.19 (0.62, 2.26)	1.14 (0.61, 2.12)
BMI ≥25 kg/m^2^				
ASM/Wt (quartiles)				
Q4	11	2 (18.2)	1.00 (reference)	1.00 (reference)
Q3	48	19 (39.6)	2.44 (0.47, 12.87)	2.62 (0.44, 14.99)
Q2	94	34 (36.2)	1.89 (0.37, 9.46)	1.71 (0.31, 9.79)
Q1	196	66 (33.7)	1.63 (0.34, 8.01)	1.44 (0.27, 8.11)
ASM/Wt (continuous)				
Per 10% decrease	349	121 (34.7)	0.66 (0.22, 2.05)	0.61 (0.19, 1.80)

Values are presented as number (%) or odds ratio (95% confidence interval). ASM/Wt, appendicular skeletal muscle mass/weight; BMI, body mass index.

1 Adjusted for age, menopause, systolic blood pressure, hemoglobin A1c, total/high-density lipoprotein cholesterol ratio, C-reactive protein, smoking, drinking, and regular exercise.

**Table 3.** Association between ASM/Wt and carotid plaque according to BMI categories

	No. of people	People with carotid plaque	Age-adjusted	Fully adjusted^1^
Men (n=595)				
BMI <25 kg/m^2^				
ASM/Wt (quartiles)				
Q4	125	21 (16.8)	1.00 (reference)	1.00 (reference)
Q3	103	18 (17.5)	1.03 (0.52, 2.07)	1.05 (0.51, 2.15)
Q2	74	16 (21.6)	1.32 (0.64, 2.74)	1.17 (0.54, 2.53)
Q1	37	15 (40.5)	2.96 (1.30, 6.77)	2.48 (1.04, 5.90)
ASM/Wt (continuous)				
Per 10% decrease	339	70 (20.7)	4.19 (1.13, 15.56)	3.27 (0.85, 12.57)
BMI ≥25 kg/m^2^				
ASM/Wt (quartiles)				
Q4	23	7 (30.4)	1.00 (reference)	1.00 (reference)
Q3	46	12 (26.1)	0.74 (0.23, 2.33)	0.52 (0.16, 1.74)
Q2	75	20 (26.7)	0.74 (0.25, 2.14)	0.53 (0.17, 1.63)
Q1	112	29 (25.9)	0.76 (0.27, 2.10)	0.41 (0.13, 1.24)
ASM/Wt (continuous)				
Per 10% decrease	256	68 (26.6)	1.39 (0.50, 3.91)	0.90 (0.32, 2.55)
Women (n=1,274)				
BMI <25 kg/m^2^				
ASM/Wt (quartiles)				
Q4	308	23 (7.5)	1.00 (reference)	1.00 (reference)
Q3	270	25 (9.3)	1.12 (0.62, 2.05)	0.90 (0.49, 1.69)
Q2	225	22 (9.8)	1.11 (0.81, 2.07)	0.88 (0.46, 1.67)
Q1	122	11 (9.0)	0.93 (0.67, 20.1)	0.70 (0.32, 1.56)
ASM/Wt (continuous)				
Per 10% decrease	925	81 (8.8)	0.77 (0.36, 1.67)	0.62 (0.29, 1.31)
BMI ≥25 kg/m^2^				
ASM/Wt (quartiles)				
Q4	11	0 (0.0)	1.00 (reference)	1.00 (reference)
Q3	48	4 (8.3)	NA	NA
Q2	94	18 (19.2)	NA	NA
Q1	196	34 (17.4)	NA	NA
ASM/Wt (continuous)				
Per 10% decrease	349	56 (16.1)	2.25 (0.48, 10.47)	2.40 (0.47, 12.18)

Values are presented as number (%) or odds ratio (95% confidence interval). ASM/Wt, appendicular skeletal muscle mass/weight; BMI, body mass index; OR, odds ratio; CI, confidence interval; NA, not applicable.

1 Adjusted for age, menopause, systolic blood pressure, hemoglobin A1c, total/high-density lipoprotein cholesterol ratio, C-reactive protein, smoking, drinking, and regular exercise.

## SUPPLEMENTARY MATERIALS

Supplementary Material 1: Table S1 is available at http://www.e-epih.org/).

Supplementary Material 2: Table S2 is available at http://www.e-epih.org/).

## References

[1] Rosenberg IH (2011). Sarcopenia: origins and clinical relevance. Clin Geriatr Med.

[2] Rolland Y, Czerwinski S, Abellan Van Kan G, Morley JE, Cesari M, Onder G (2008). Sarcopenia: its assessment, etiology, pathogenesis, consequences and future perspectives. J Nutr Health Aging.

[3] Chung JY, Kang HT, Lee DC, Lee HR, Lee YJ (2013). Body composition and its association with cardiometabolic risk factors in the elderly: a focus on sarcopenic obesity. Arch Gerontol Geriatr.

[4] Atkins JL, Whincup PH, Morris RW, Lennon LT, Papacosta O, Wannamethee SG (2014). Sarcopenic obesity and risk of cardiovascular disease and mortality: a population-based cohort study of older men. J Am Geriatr Soc.

[5] Byeon CH, Kang KY, Kang SH, Bae EJ (2015). Sarcopenia is associated with Framingham risk score in the Korean population: Korean National Health and Nutrition Examination Survey (KNHANES) 2010-2011. J Geriatr Cardiol.

[6] Hwang YC, Cho IJ, Jeong IK, Ahn KJ, Chung HY (2017). Differential association between sarcopenia and metabolic phenotype in Korean young and older adults with and without obesity. Obesity (Silver Spring).

[7] Chin SO, Rhee SY, Chon S, Hwang YC, Jeong IK, Oh S (2013). Sarcopenia is independently associated with cardiovascular disease in older Korean adults: the Korea National Health and Nutrition Examination Survey (KNHANES) from 2009. PLoS One.

[8] Kim TN, Park MS, Lim KI, Yang SJ, Yoo HJ, Kang HJ (2011). Skeletal muscle mass to visceral fat area ratio is associated with metabolic syndrome and arterial stiffness: the Korean Sarcopenic Obesity Study (KSOS). Diabetes Res Clin Pract.

[9] Shim JS, Song BM, Lee JH, Lee SW, Park JH, Choi DP (2017). Cardiovascular and Metabolic Diseases Etiology Research Center (CMERC) cohort: study protocol and results of the first 3 years of enrollment. Epidemiol Health.

[10] Furushima T, Miyachi M, Iemitsu M, Murakami H, Kawano H, Gando Y (2017). Comparison between clinical significance of height-adjusted and weight-adjusted appendicular skeletal muscle mass. J Physiol Anthropol.

[11] Meng NH, Li CI, Liu CS, Lin CH, Lin WY, Chang CK (2015). Comparison of height- and weight-adjusted sarcopenia in a Taiwanese metropolitan older population. Geriatr Gerontol Int.

[12] Abbatecola AM, Chiodini P, Gallo C, Lakatta E, Sutton-Tyrrell K, Tylavsky FA (2012). Pulse wave velocity is associated with muscle mass decline: Health ABC study. Age (Dordr).

[13] Campos AM, Moura FA, Santos SN, Freitas WM, Sposito AC; Brasilia Study on Healthy Aging and Brasilia Heart Study (2017). Sarcopenia, but not excess weight or increased caloric intake, is associated with coronary subclinical atherosclerosis in the very elderly. Atherosclerosis.

[14] Kato A, Ishida J, Endo Y, Takita T, Furuhashi M, Maruyama Y (2011). Association of abdominal visceral adiposity and thigh sarcopenia with changes of arteriosclerosis in haemodialysis patients. Nephrol Dial Transplant.

[15] Ochi M, Kohara K, Tabara Y, Kido T, Uetani E, Ochi N (2010). Arterial stiffness is associated with low thigh muscle mass in middle-aged to elderly men. Atherosclerosis.

[16] Jensky NE, Allison MA, Loomba R, Carnethon MR, de Boer IH, Budoff MJ (2013). Null association between abdominal muscle and calcified atherosclerosis in community-living persons without clinical cardiovascular disease: the multi-ethnic study of atherosclerosis. Metabolism.

[17] Wassel CL, Laughlin GA, Saad SD, Araneta MR, Wooten W, Barrett-Connor E (2015). Associations of abdominal muscle area with 4-year change in coronary artery calcium differ by ethnicity among post-menopausal women. Ethn Dis.

[18] Aubertin-Leheudre M, Lord C, Goulet ED, Khalil A, Dionne IJ (2006). Effect of sarcopenia on cardiovascular disease risk factors in obese postmenopausal women. Obesity (Silver Spring).

[19] Soisson V, Brailly-Tabard S, Empana JP, Féart C, Ryan J, Bertrand M (2012). Low plasma testosterone and elevated carotid intima-media thickness: importance of low-grade inflammation in elderly men. Atherosclerosis.

[20] Kohara K, Ochi M, Tabara Y, Nagai T, Igase M, Miki T (2012). Arterial stiffness in sarcopenic visceral obesity in the elderly: J-SHIPP study. Int J Cardiol.

[21] Balage M, Averous J, Rémond D, Bos C, Pujos-Guillot E, Papet I (2010). Presence of low-grade inflammation impaired postprandial stimulation of muscle protein synthesis in old rats. J Nutr Biochem.

[22] Savetsky IL, Torrisi JS, Cuzzone DA, Ghanta S, Albano NJ, Gardenier JC (2014). Obesity increases inflammation and impairs lymphatic function in a mouse model of lymphedema. Am J Physiol Heart Circ Physiol.

[23] Rocha VZ, Libby P (2009). Obesity, inflammation, and atherosclerosis. Nat Rev Cardiol.

[24] Abe T, Thiebaud RS, Loenneke JP, Bemben MG, Loftin M, Fukunaga T (2012). Influence of severe sarcopenia on cardiovascular risk factors in nonobese men. Metab Syndr Relat Disord.

[25] Dey DK, Bosaeus I, Lissner L, Steen B (2009). Changes in body composition and its relation to muscle strength in 75-year-old men and women: a 5-year prospective follow-up study of the NORA cohort in Göteborg, Sweden. Nutrition.

[26] Xue B, Johnson AK, Hay M (2013). Sex differences in angiotensin II- and aldosterone-induced hypertension: the central protective effects of estrogen. Am J Physiol Regul Integr Comp Physiol.

[27] Sandberg K, Ji H (2012). Sex differences in primary hypertension. Biol Sex Differ.

[28] Stewart SP, Bramley PN, Heighton R, Green JH, Horsman A, Losowsky MS (1993). Estimation of body composition from bioelectrical impedance of body segments: comparison with dual-energy X-ray absorptiometry. Br J Nutr.

[29] Boneva-Asiova Z, Boyanov MA (2008). Body composition analysis by leg-to-leg bioelectrical impedance and dual-energy X-ray absorptiometry in non-obese and obese individuals. Diabetes Obes Metab.

[30] Malavolti M, Mussi C, Poli M, Fantuzzi AL, Salvioli G, Battistini N (2003). Cross-calibration of eight-polar bioelectrical impedance analysis versus dual-energy X-ray absorptiometry for the assessment of total and appendicular body composition in healthy subjects aged 21-82 years. Ann Hum Biol.

