# Supplementary material for: Association of appendicular skeletal muscle mass with carotid intima-media thickness according to body mass index in Korean adults
Source: Epidemiol Health. 2018 Oct 7;40:e2018049. doi: 10.4178/epih.e2018049 (PMC6288657; doi:10.4178/epih.e2018049)
Supplement: Supplementary file 1 [file epih-40-e2018049-supplementary1.pdf]

Supplementary Materials

Table S1. Characteristics of the study participants in total and by sex

Variables	Total (n=1,869)	Men (n=595)	Women (n=1,274)	*p* value
Age, years	54.3 ± 6.2	54.6 ±6.6	54.2 ±6.0	0.248
Height, cm	161.0 ± 7.9	169.5 ±5.8	157.0 ±5.1	<0.001
Weight, kg	62.3 ± 10.0	70.8 ±9.5	58.3 ±7.5	<0.001
BMI, kg/m^2	23.9 ± 2.8	24.6 ±2.7	23.6 ±2.8	<0.001
Waist circumference, cm	80.9 ± 8.5	86.0 ±7.5	78.4 ±7.8	<0.001
ASM, kg	18.2 ± 4.0	22.9 ±3.0	15.9 ±2.1	<0.001
ASM/Wt, %	29.1 ± 3.6	32.5 ±2.8	27.5 ±2.7	<0.001
Systolic BP, mmHg	118.6 ± 14.8	124.9 ±14.3	115.7 ±14.0	<0.001
Diastolic BP, mmHg	75.6 ± 9.7	80.3 ±9.9	73.4 ±8.8	<0.001
TC, mg/dL	199.9 ± 34.8	195.5 ±34.5	201.9 ±34.7	<0.001
HDLC, mg/dL	57.1 ± 14.3	51.5 ±12.8	59.8 ±14.1	<0.001
LDLC, mg/dL	120.4 ± 31.0	120.1 ±31.6	120.6 ±30.7	0.798
TG, mg/dL	109 [80-150]	128 [91-179]	103 [76-136]	<0.001
TC/HDLC	3.7 ± 0.9	4.0 ±1.0	3.5 ±0.9	<0.001
Fasting glucose, mg/dL	89 [83-96]	92 [85-101]	88 [82-94]	<0.001
Hemoglobin A1c, %	5.7 ± 0.7	5.8 ±0.8	5.7 ±0.7	0.228
C-reactive protein, mg/L	0.6 [0.3-1.2]	0.7 [0.4-1.4]	0.6 [0.3-1.2]	0.005
IMT, mm	0.666 ± 0.103	0.682 ±0.107	0.659 ±0.101	<0.001
Highest quartile of IMT	471 (25.2)	150 (25.2)	321 (25.2)	0.995
Carotid plaque	275 (14.7)	138 (23.2)	137 (10.8)	<0.001
Antihypertensive medication	341 (18.3)	134 (22.5)	207 (16.3)	0.001
Antidiabetic medication	95 (5.1)	45 (7.6)	50 (3.9)	<0.001
Lipid-lowering medication	244 (13.1)	61 (10.3)	183 (14.4)	0.014
Smoking status				
Non-smoker	1,344 (71.9)	137 (23.0)	1,207 (94.7)	
Former smoker	322 (17.2)	284 (47.7)	38 (3.0)	<0.001
Current smoker	203 (10.9)	174 (29.3)	29 (2.3)	
Drinking status				
Non-drinker	572 (30.6)	67 (11.3)	505 (39.6)	
Former drinker	84 (4.5)	43 (7.2)	41 (3.2)	<0.001
Current regular drinker	1,213 (64.9)	485 (81.5)	728 (57.2)	
Regular exercise				
Yes	1,180 (63.1)	386 (64.9)	794 (62.3)	0.287
No	689 (36.9)	209 (35.1)	480 (37.7)	
Sleep duration, h/day	6.9 ± 1.2	7.0 ±1.2	6.8 ±1.2	0.006

Data are expressed as means ± standard deviations, medians [interquartile ranges], or numbers (percentages).
The *p* values were derived using an independent t-test, Wilcoxon rank-sum test, or chi-square test.
BMI: body mass index, ASM/Wt: appendicular skeletal muscle mass/weight, BP: blood pressure, TC: total cholesterol, HDLC: high-density lipoprotein cholesterol, LDLC: low-density lipoprotein cholesterol, TG: triglycerides, IMT: intima-media thickness
