# Supplementary material for: Association of appendicular skeletal muscle mass with carotid intima-media thickness according to body mass index in Korean adults
Source: Epidemiol Health. 2018 Oct 7;40:e2018049. doi: 10.4178/epih.e2018049 (PMC6288657; doi:10.4178/epih.e2018049)
Supplement: Supplementary file 2 [file epih-40-e2018049-supplementary2.pdf]

Table S2. Interactions between ASM/Wt quartiles and BMI categories in the presence of highest quartile of IMT

	BMI categories		*p* for interaction
	<25 kg/m^2	≥25 kg/m^2	
Men (n=595)			
ASM/Wt quartiles			
Q4	Reference	Reference	0.016
Q3	1.81 (0.87 - 3.74)	1.06 (0.37 - 3.02)	
Q2	2.17 (1.01 - 4.65)	0.68 (0.23 - 1.77)	
Q1	4.71 (1.99 - 11.13)	1.04 (0.41 - 2.67)	
Women (n=1,274)			
ASM/Wt quartiles			
Q4	Reference	Reference	0.707
Q3	1.23 (0.82 - 1.86)	2.95 (0.57 - 15.16)	
Q2	1.49 (0.98 - 2.27)	2.55 (0.52 - 12.49)	
Q1	1.47 (0.89 - 2.43)	2.29 (0.48 - 10.88)	

Data are expressed as odds ratios (95% confidence intervals) for highest quartile of IMT.

ASM/Wt: appendicular skeletal muscle mass/weight, BMI: body mass index, IMT: intima-media thickness
